# The impact of obsessive-compulsive personality disorder on obsessive-compulsive disorder: clinical outcomes in the context of bipolarity

**DOI:** 10.3389/fpsyt.2025.1532966

**Published:** 2025-04-24

**Authors:** Maciej Żerdziński, Marcin Burdzik, Paweł Dębski, Roksana Żmuda, Magdalena Piegza, Piotr Gorczyca

**Affiliations:** ^1^ Psychiatric Department No 2, Dr. Krzysztof Czuma’s Psychiatric Center, Katowice, Poland; ^2^ Department of Psychiatry and Sexology, Faculty of Medicine, Academy of Silesia, Katowice, Poland; ^3^ Institute of Law at Faculty of Law and Administration, University of Silesia in Katowice, Katowice, Poland; ^4^ Institute of Psychology, Humanitas University in Sosnowiec, Sosnowiec, Poland; ^5^ Department of Psychiatry, Faculty of Medical Sciences in Zabrze, Medical University of Silesia in Katowice, Tarnowskie Gory, Poland

**Keywords:** OCD, OCPD, OCD comorbidity, OCPD comorbidity, bipolarity, BD predictors, MDD predictors

## Abstract

Obsessive-compulsive disorder (OCD) is characterized by obsessions and compulsions that significantly impair functioning. Obsessive-compulsive personality disorder (OCPD) co-occurs in 17-45% of OCD patients, worsening outcomes across multiple domains. Therefore, we aimed to study the impact of OCPD in more detail by analyzing selected comorbidities, emotional aspects, and sociodemographic data. This study assessed 78 OCD patients (average age 44.9 years, 34.61% OCPD), using Y-BOCS, BABS, BPAQ, BIS-11, YMRS, HDRS-17, and ASEX. Patients with comorbid OCPD had significantly worse outcomes in symptom severity (Y-BOCS = 0.0006), treatment duration (p = 0.0127), insight (BABS, p = 0.0185), aggression (p = 0.0266), impulsivity (p = 0.0469), depression (HDRS, p = 0.0178), mania (YMRS, p = 0.0003), and sexual dysfunction (ASEX, p = 0.008). OCPD was more prevalent in unemployed individuals (p = 0.046) and older patients (p = 0.009). No significant differences were found regarding gender, education, or relationship status. Obsessions and compulsions, such as contamination (p = 0.025), somatic (p = 0.018), ruminations (p = 0.003), and obsessional slowness (p = 0.007), were more common in the OCPD group. In the group with OCPD, aggression and OCD severity were correlated with increased levels of depression, which can be considered potential correlates of bipolarity in the relationship between OCD and OCPD. In conclusion, OCPD significantly worsens clinical outcomes in OCD across emotional, behavioral, and functional dimensions.

## 1 Introduction

### 1.1 OCD

Obsessive-compulsive disorder (OCD) is characterized by the presence of both obsessions and compulsions (OC). Obsessions are defined as recurrent, unwanted thoughts, impulses, or imaginings, most often perceived as intrusive, while compulsions refer to pathological motor activities or mental acts (1–7). Many researchers suggest that the emotional system is also connected to the phenomenology of OCD, especially in relation to anxiety and anger (8–11). Epidemiological studies estimate the prevalence of OCD to be between 2-3, 5% (12, 13). The initiation of therapy for OCD is often delayed and remains unsatisfactory in terms of efficacy (14, 15). Longitudinal studies report varying remission rates, ranging from 12% to 86% (16–18). The literature indicates that even after satisfactory treatment, 40-60% of OCD patients continue to experience disabling residual symptoms (19–21). In severe and chronic cases, even after significant improvement in OC, patients may continue to struggle with adjusting to normal life, a phenomenon referred to as the ‘burden of normality’ (22). This difficulty can be partly attributed to increased aggression and impulsivity, as well as frequent comorbidities, including depression (MD), bipolar disorder (BD), and obsessive-compulsive personality disorder (OCDP) (23–28).

### 1.2 Obsessive-compulsive personality disorder

OCPD (anankastic personality) is a distinct clinical phenomenon within the obsessive-compulsive spectrum (29, 30). The term ‘anankastic personality’ is rooted in Greek mythology, where Ananke, the goddess of necessity, symbolized inevitability, compulsion, and submission to fate. This term aptly conveys the relentless need for control and the strength of character associated with the concept (31–33). Psychoanalysts concur that the need for control in individuals with OCPD functions as a defense mechanism, concealing deeper desires for dependence rooted in childhood. The anxiety resulting from a perceived loss of control creates a barrier to emotional intimacy (34). This rigidity leads to resistance to new experiences and is reflected in traits such as low agreeableness, overzealousness, and heightened sensitivity to criticism (35–37). As a result, these individuals often exhibit antagonistic behaviors, show increased aggression and impulsivity, struggle with trust, and have difficulty maintaining interpersonal harmony, which may foster tendencies toward dominance or even tyranny (31, 33, 35, 38–43).

In both ICD-10 (F60.5) and DSM-5 (301.4), OCPD is characterized by a pervasive preoccupation with order, rules, and details, often leading to inefficiency in daily life. This rigid focus on perfectionism and control causes individuals to adhere strictly to procedures and routines, limiting their ability to adapt and hindering productivity. Several key traits in OCPD contribute to interpersonal conflicts. First, individuals with OCPD tend to be excessively conscientious and morally scrupulous, frequently imposing their rigid standards on others. This inflexibility leads to tension, particularly in professional and personal environments. Moreover, their extreme reluctance to delegate tasks unless others follow their exact methods is perceived as controlling, exacerbating relational strain. As these individuals push their standards onto others, they often fail to recognize the negative impact on their relationships, leading to a pattern of isolation and increasing emotional distress. This ongoing tension reinforces their need for control, perpetuating a cycle of conflict and emotional dissatisfaction that ultimately worsens their mental health (2, 31, 33, 40, 41, 44).

In ICD-11, the concept of anankastia (6D11.4) represents a shift towards a dimensional approach to diagnosing personality disorders, focusing on traits like perfectionism, control, and inflexibility. This model assesses the severity of these traits within specific domains of personality dysfunction. The characteristics of anankastia are largely consistent with descriptions found in ICD-10 and DSM-5, which emphasize similar patterns of rigidity, emotional constraint, and overemphasis on order and rules (44–46). Some researchers have considered including OCPD in the category of Obsessive-Compulsive and Related Disorders (OCRD) due to shared traits such as rigidity, perfectionism, and excessive control and orderliness. Although the etiological factors for these disorders may differ, the shared comorbidities in OCPD strengthen the proposal for this reclassification (29, 47–49).

The prevalence of OCPD in the general population ranges from 3-8%, with a global estimate of 6.5% based on a meta-analysis conducted by Clemento et al. (50).

OCPD is more common in older, less educated individuals, and in clinical populations, the prevalence varies, ranging from 8.7% to 26% among outpatients, and reaching 23.3% in psychiatric inpatient settings (33, 41, 50–52).

### 1.3 OCD and OCPD comorbidity

OCPD is thought to occur in 15-36% of individuals with OCD and is generally associated with a worse course and less favorable prognosis (29, 30, 42, 53–56). This correlation indicates an earlier age of OCD onset, stronger OC severity, poorer insight, higher comorbidity with depression and anxiety, and greater impairment in functioning (57). Patients with both OCD and OCPD are more likely to exhibit symptoms related to hoarding, symmetry, ordering, doubting, checking, counting, and arranging (55, 58). Unlike the ego-dystonic obsessions found in OCD, the obsessions in OCPD (if present) are ego-syntonic and align with the patient’s beliefs, meaning they are not necessarily perceived as unhealthy, although they can cause significant distress to others (59, 60). While there are similarities between OCPD and OCD, the later development of obsessive-compulsive symptoms should not be viewed as a direct extension of anankastic character traits (29, 48, 49).

### 1.4 OCD, OCPD, and affective disorders: understanding the links

The relationships between OCD and MDD, as well as BD, are well-researched. However, the link between OCPD and these conditions - particularly BD - remains less thoroughly explored.

#### 1.4.1 OCD and MDD

OCD and MDD frequently co-occur, with 20-50% of individuals diagnosed with OCD and 20-30% of those with MDD experiencing both disorders (26, 61–63). Epidemiological studies report that the lifetime prevalence of MDD in OCD patients ranges from 40% to 67%, with OCD often preceding depressive symptoms, particularly in early-onset cases (26, 27, 64–67). Additionally, the presence of depression significantly reduces the quality of life in patients with OCD (68). MDD can also delay and complicate the treatment of OCD, with a dual diagnosis being associated with an elevated risk of suicide attempts in 6% to 52% of affected patients (17, 21, 69, 70). Chaudhary et al. (71) studied patients with both depression and OCD, demonstrating that suicidal ideation was present in 52% of the sample, with 16% having a history of suicide attempts. It was also shown that the severity of suicidal ideation correlated with the intensity of depressive symptoms, with suicidal ideation occurring in all cases of severe and very severe depression co-occurring with OCD, compared to 35% in mild and 87.5% in moderate depression. Suicidal thoughts were most commonly associated with cleanliness/contamination and religious obsessions (71). Following the recent study by Hellberg et al. (72), the presence of MDD in OCD predicts poorer treatment outcomes, with severe depression most strongly associated with repugnant or taboo obsessions. Weaker or absent correlations are observed between depression and other OCD dimensions, such as contamination-related obsessions (72).

#### 1.4.2 OCD and BD

The links between OCD and BD have solid historical grounding. In 1921, Emil Kraepelin described cases of ‘anxious mania’ and ‘agitated depression’, highlighting anxiety as a key feature of BD. Around the same period, Abraham and Gero (1921, 1936) identified that individuals in manic episodes often engage in obsessive thinking, especially in relational contexts, where their obsession tends to focus on the object of desire (73, 74). Subsequent studies have confirmed the observation that OC frequently emerge during mixed episodes in BD, often linked with more frequent depressive recurrences (73, 75, 76). Hantouche et al. (77) identified a specific diagnostic subcategory within the group of patients suffering from OCD: ‘Cyclothymic-OCD’. In this subgroup, mood decompensation - both depressive and hypomanic - was particularly common, with OC being more episodic (77). Research indicates that the prevalence of OCD in BD patients ranges from 11% to 35.2%, while lifetime comorbidity rates of BD among OCD patients range from 11.1% to 21%. Additionally, OCD is more commonly observed in children and adolescents with BD compared to adults, and is more frequently reported in population-based studies than in hospital-based ones (7, 28, 73, 78).

The meta-analysis by Amerio et al. (79) reinforces the relationship between OCD and BD, showing that the pooled prevalence of OCD in BD patients is 17%, which is comparable to the prevalence of BD in OCD patients (18.35%). The study also highlights that OCD is more prevalent in BD-I patients (24.6%) compared to mixed BD patients (13.6%), indicating a stronger comorbidity with the more severe form of bipolar disorder (79). Notably, it has been demonstrated that 35% of BD patients in remission exhibit OCD symptoms, suggesting a higher prevalence in this group compared to those with active symptoms (80). It was also observed that among OCD patients, 53.9% were diagnosed with a dominant cyclothymic affective temperament, which was the most represented in the sample (19.2%) (81). OCD-BD was characterized by a more chronic course, higher dysfunction, suicide and hostility. In turn, OC aggressive symptoms, having first-degree relatives with OCD and comorbidity of any anxiety disorders were associated with a reduction in odds of belonging to the OCD-BD group (82). Individuals with bipolar OCD exhibit higher rates of sexual and religious obsessions and lower rates of checking rituals compared to those with non-bipolar OCD. Studies have also shown an association between symmetry obsessions, repetition, and ordering compulsions (83). Sharma and Reddy (73), in their review of the literature on the comorbidity of OCD and BD, highlighted the inconsistency of findings regarding OCS in this patient group. BD combined with OCD was associated with more frequent sexual, religious, aggressive, symmetry, and hoarding obsessions, as well as ordering and hoarding compulsions. However, other studies reported that contamination obsessions and washing compulsions were less common. There was no evidence of more severe OCS in this group, but BD-OCD patients exhibited poorer insight into OCD and experienced more frequent depressive episodes and suicidal thoughts. Additionally, studies showed higher rates of substance abuse and comorbid anxiety disorders, ADHD, and personality disorders in BD-OCD patients (73).

#### 1.4.3 OCPD and MDD

The relationship between OCPD and MDD has been studied more frequently then BD with several findings suggesting that the presence of OCPD traits, particularly perfectionism and high moral standards, significantly increases the recurrence of depressive episodes and the risk of suicide (32, 84, 85). It has also been demonstrated that the presence of OCPD associated with MDD may increase the risk of misdiagnosis, make certain life events more stressful than usual for patients, and, once again, result in an elevated risk of suicide (86). Van Broekhoven et al. (87) highlighted the greater susceptibility of women with OCPD to postpartum depression, emphasizing the need for careful assessment of anankastic personality traits in pregnant women to better monitor and mitigate the risks of depression and suicide (87).

#### 1.4.4 OCPD and BD

The relationship between OCPD and BD has remained significantly understudied. Rossi et al. (87) found that anankastic personality traits were the most frequently co-diagnosed in patients with BD, with a prevalence of 32.4%, compared to 31.6% for dependent personality traits in patients with recurrent depressive disorders (88). Similarly, Altindag (88) reported that OCPD was the most common personality disorder (21%) among patients with BD type I (89). It has also been described that when OCPD co-occurs with OCD, mood instability may exacerbate the presence of OCPD traits. These findings suggest a potentially significant yet underrecognized role of OCPD in influencing the course of OCD in the context of BD, underscoring the need for further research into this complex clinical relationship (90).

When considering the relationships between OCD, OCPD, and BD independently, it is important to note a significant lack of research addressing the impact of OCPD on OCD in the context of its comorbidity with BD.

## 2 Objectives

- Assess the prevalence of OCPD in individuals with OCD.
- Investigate the associations between OCPD and selected sociodemographic variables, particularly age, as well as factors such as employment status, the ability to maintain close relationships, and sexual functioning.
- Evaluate the impact of OCPD on the clinical course of OCD by examining selected psychopathological factors associated with worse prognosis, including OCD severity, reduced insight, aggression, impulsivity, and the occurrence of depression and mania.
- Identify the most prevalent OC symptoms in patients with coexisting OCPD.
- Analyze statistically significant findings in the OCD with OCPD group to explore their associations with levels of depression and mania, focusing on the role of bipolarity within the context of OCPD’s influence on OCD.

## 3 Materials and methods

This study involved seventy-eight patients with a confirmed primary diagnosis of obsessive-compulsive disorder (OCD). Participants were selected from the hospital outpatient clinic based on their diagnosis, with no additional selection criteria applied. Although the researchers were affiliated with various academic and hospital institutions, the study was conducted exclusively at a single center, the Dr. Krzysztof Czuma Psychiatric Center in Katowice, Poland. Informed consent was obtained from each participant prior to examination and inclusion in the study. All investigators had extensive experience in managing OCD patients. The study procedures were approved by the local bioethics committee (approval number BNW/NWN/0052/KB/172/23).

The current mental state of all subjects was evaluated using the following validated diagnostic questionnaires:

- OCD assessment (presence and severity): Yale-Brown Obsessive-Compulsive Scale (Y-BOCS) (91),
- Assessment of OCD level of insight: Brown Assessment of Beliefs Scale (BABS) (92),
- Assessment of aggression level: Buss-Perry Aggression Questionnaire (BPAQ) (93),
- Assessment of impulsivity level: Barratt Impulsivity Scale (BIS-11) (94),
- Assessment of the presence of affective symptoms: Young Mania Rating Scale (YMRS) (95) and Hamilton Depression Rating Scale (HDRS-17) (96),
- Sexual functioning evaluation: Arizona Sexual Experiences Scale (ASEX) (97),
- The duration of OCD treatment was assessed based on medical records and medical history collected from the subjects,
- Identification of OCPD traits was conducted according to the DSM-5 criteria (2). These traits had to be chronic, begin in early adulthood, and persist regardless of circumstances.

Based on the obtained results, the total study group was divided into two subgroups: patients with OCD without OCPD and patients with both OCD and OCPD. These subgroups were compared across the following variables:

- age, gender, education, employment status, ability to maintain close relationships, and sexual functioning
- OCD severity, levels of insight, characteristics of obsessive-compulsive symptoms, and duration of treatment
- levels of aggression, impulsivity, depression, and mania

Additionally, an analysis was conducted to determine which of the statistically significant findings correlated with levels of depression and mania.

Data processing was conducted using Excel 2016 and Statistica version 13.3. The normality of the distribution of variables was verified with the Shapiro-Wilk test. Skewness and kurtosis tests were used to assess the data distribution for asymmetry and kurtosis relative to a normal distribution. The Mann-Whitney U test was used to assess the significance of differences in continuous variables between patients with OCD and patients with both OCD and OCPD. The association between the co-occurrence of OCD and OCPD with individual obsessions and compulsions was assessed using the chi-square test. The level of statistical significance was set at α ≤ 0.05.

## 4 Results

Among the study participants, 36 (46.15%) were men and 42 (53.84%) were women. The average age of the respondents was 44.9 years. Regarding education, 57.69% (n = 45) of respondents had a university degree, while 42.31% (n = 33) had lower levels of education (secondary, vocational, primary). A total of 65.38% (n = 51) of individuals were in a close relationship, while 34.62% (n = 27) were single. Additionally, 61.54% of subjects (n = 48) were employed, and 38.46% (n = 30) were unemployed.

It was found that in the entire study group (n = 78), 34.61% of participants (n = 27) met the criteria for OCPD, while 65.38% (n = 51) did not. According to the chi-square test results, there were no significant differences in the prevalence of OCPD among OCD patients when considering gender (chi-square = 0.553; p = 0.457), education level (chi-square = 2.207; p = 0.137), or relationship status (chi-square = 0.037; p = 0.847). However, OCPD was found to be significantly more common among the unemployed (67.74%) compared to the employed (44.90%) (chi-square = 3.986; p = 0.046). It was also observed that participants with both OCD and OCPD were significantly older (median = 48) compared to those with OCD alone (median = 38) (p = 0.009). Descriptive statistics of continuous variables are presented in **Table 1** for patients with OCD and those with both OCD and OCPD.

**Table 1:** Descriptive statistics of variables in the group of patients with OCD without OCPD (n = 51) and with OCD and OCPD (n = 27).

	OCD without OCPD (n = 51)						OCD with OCPD (n = 27)					
	Mean	SD	Min	Max	Skewness	Kurtosis	Mean	SD	Min	Max	Skewness	Kurtosis
OCD T	7.235	6.819	0	25	0.887	-0.036	13.185	10.348	0	35	0.42	-0.874
Y-BOCS	20.725	6.876	8	32	-0.120	-1.081	26.926	5.512	17	38	0.335	-0.653
Aggression	75.745	18.363	41	118	0.078	-0.451	85.444	15.793	58	126	0.465	0.288
Impulsivity	61.863	10.323	41	90	0.734	0.694	67.222	12.22	46	96	0.44	0.027
YMRS	2.569	4.168	0	24	3.548	15.239	5.741	4.785	0	20	1.147	1.571
HDRS	7.608	5.514	0	29	1.297	3.157	11.038	6.89	3	36	1.928	5.892
BABS	1.333	1.071	0	4	0.708	0.386	1.852	1.027	0	4	-0.373	0.048
ASEX	21.762	6.525	7	30	0.641	0.290	21.762	6.525	5	30	-0.391	-0.569

OCD T, duration of OCD treatment (in years); Y-BOCS, Yale-Brown Obsessive-Compulsive Scale result; Aggression, Buss-Perry Aggression Questionnaire (total score); Impulsivity, Barratt Impulsivity Scale (total score); YMRS, Young Mania Rating Scale result; HDRS, Hamilton Depression Rating Scale result; BABS, Brown Assessment of Beliefs Scale result; ASEX, Arizona Sexual Experiences Scale result.

**Figure 1:**
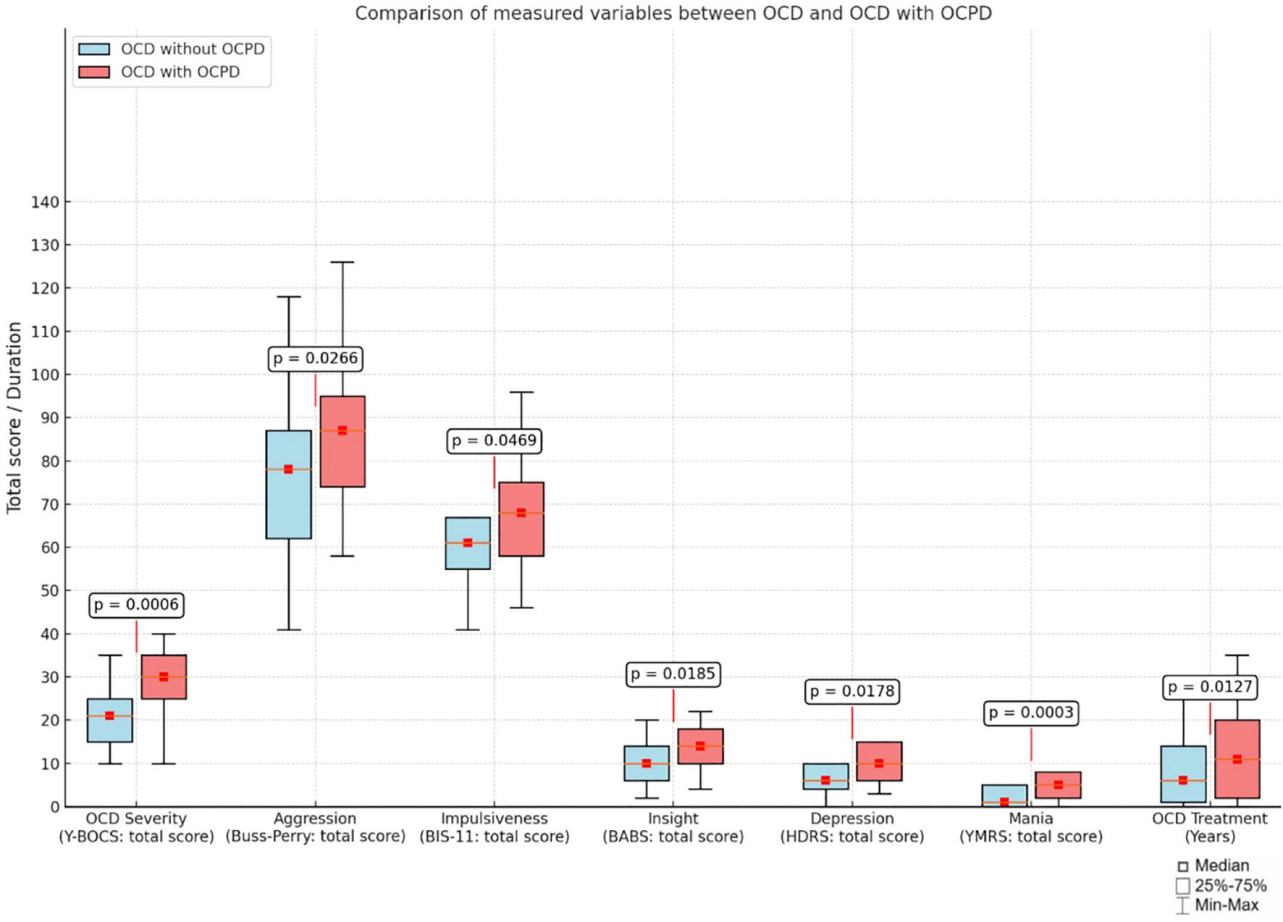
Comparison of continuous variables between patients with OCD without OCPD and patients with both OCD and OCPD.

Significance analysis of differences between OCD and OCPD patients using the Mann-Whitney U test showed statistically significant differences between subgroups in Y-BOCS, BPAQ, BIS-11, BABS, YMRS, HDRS, and ASEX scores. In all parameters, significantly higher values were obtained in patients with both OCD and OCPD. This means that those with a dual diagnosis were characterized by higher severity of OCD symptoms, higher levels of aggression and impulsivity, poorer levels of insight, and higher severity of both depression and mania. Patients with OCD and OCPD showed significantly worse sexual functioning as measured by the ASEX scale. This group of patients also had a statistically significant longer duration of treatment than patients with isolated OCD. Differences in the severity of individual variables are shown in **Table 2** and **Figure 1**.

**Table 2:** Differences in severity of continuous variables between patients with OCD without OCPD and patients with OCD and OCPD.

	OCD (n = 51)			OCD with OCPD (n = 27)			Mann-Whitney U	
	Median	QR		Median	QR		Z	p
OCD T	6.000	1.000	11.000	14.000	2.000	20.000	-2.4915	**0.0127**
Y-BOCS	21.000	14.000	27.000	25.000	24.000	32.000	-3.4502	**0.0006**
Aggression	78.000	62.000	87.000	87.000	74.000	95.000	-2.2170	**0.0266**
Impulsivity	61.000	55.000	68.000	67.000	58.000	75.000	-1.9869	**0.0469**
YMRS	1.000	0.000	3.000	5.000	2.000	8.000	-3.5939	**0.0003**
HDRS	6.000	4.000	10.000	10.000	6.000	15.000	-2.3708	**0.0178**
BABS	1.000	1.000	2.000	2.000	1.000	2.000	-2.3562	**0.0185**
ASEX	17.000	13.000	20.000	21,500	18.000	28.000	-2,637	**0,008**

OCD T, duration of OCD treatment (in years); Y-BOCS, general severity of OCD; Aggression, Buss-Perry Scale general result; Impulsivity, general intensity of impulsivity; YMRS, Young Mania Rating Scale result; HDRS, Hamilton Depression Scale result; BABS, Brown Assessment of Beliefs Scale result; ASEX, Arizona Sexual Experiences Scale result.

Bolded values indicate statistically significant results.

In assessing the strength of the relationships between variables that were found to be statistically significant for the OCD with OCPD group and levels of mania and depression, statistically significant and positive correlations were found between HDRS and BP-total (p = 0.353), BP-A (p = 0.400), and BP-H (p = 0.322). Additionally, positive correlations were observed between Y-BOCS total (p = 0.421), Y-BOCS obsessions (p = 0.378), and Y-BOCS compulsions (p = 0.423) with HDRS. The following variables did not show statistically significant correlations with either YMRS or HDRS: age (p = 0.035 for YMRS, p = 0.083 for HDRS), BP-PA (p = 0.160 for YMRS, p = 0.088 for HDRS), BP-VA (p = 0.035 for YMRS, p = 0.262 for HDRS), impulsiveness (p = 0.044 for YMRS, p = 0.082 for HDRS), cognitive impulsivity (p = 0.022 for YMRS, p = 0.238 for HDRS), motor impulsivity (p = 0.177 for YMRS, p = -0.011 for HDRS), planning impulsivity (p = -0.044 for YMRS, p = 0.073 for HDRS), BABS (p = 0.081 for YMRS, p = -0.073 for HDRS), and treatment length (p = -0.087 for YMRS, p = 0.064 for HDRS). **Table 3** presents Spearman correlation coefficients between selected clinical variables and the YMRS and HDRS in the group of patients with OCD and OCPD.

**Table 3:** Spearman correlation coefficients for the group of individuals with OCD and OCPD (n = 27).

	YMRS	HDRS
Age	-0,035	0,083
Treatment length	-0,087	0,064
BP-total	0,176	0,353*
BP-PA	0,160	0,088
BP-VA	0,035	0,262
BP-A	0,165	0,400*
BP-H	0,134	0,322*
Impulsiveness	0,044	0,082
Cognitive impulsivity	0,022	0,238
Motor impulsivity	0,177	-0,011
Planning impulsivity	-0,044	0,073
Y-BOCS total	0,270	0,421*
Y-BOCS obsessions	0,222	0,378*
Y-BOCS compulsions	0,242	0,423*
BABS	0,081	-0,073

OCD T, duration of OCD treatment (in years); Y-BOCS, Yale-Brown Obsessive-Compulsive Scale result; Aggression, Buss-Perry Aggression Questionnaire (total score); Impulsivity, Barratt Impulsivity Scale (total score); YMRS, Young Mania Rating Scale result; HDRS, Hamilton Depression Rating Scale result; BABS, Brown Assessment of Beliefs Scale result; ASEX, Arizona Sexual Experiences Scale result. * statistically significant at p ≤ 0.05.

Bolded values indicate statistically significant results.

**Figure 2:**
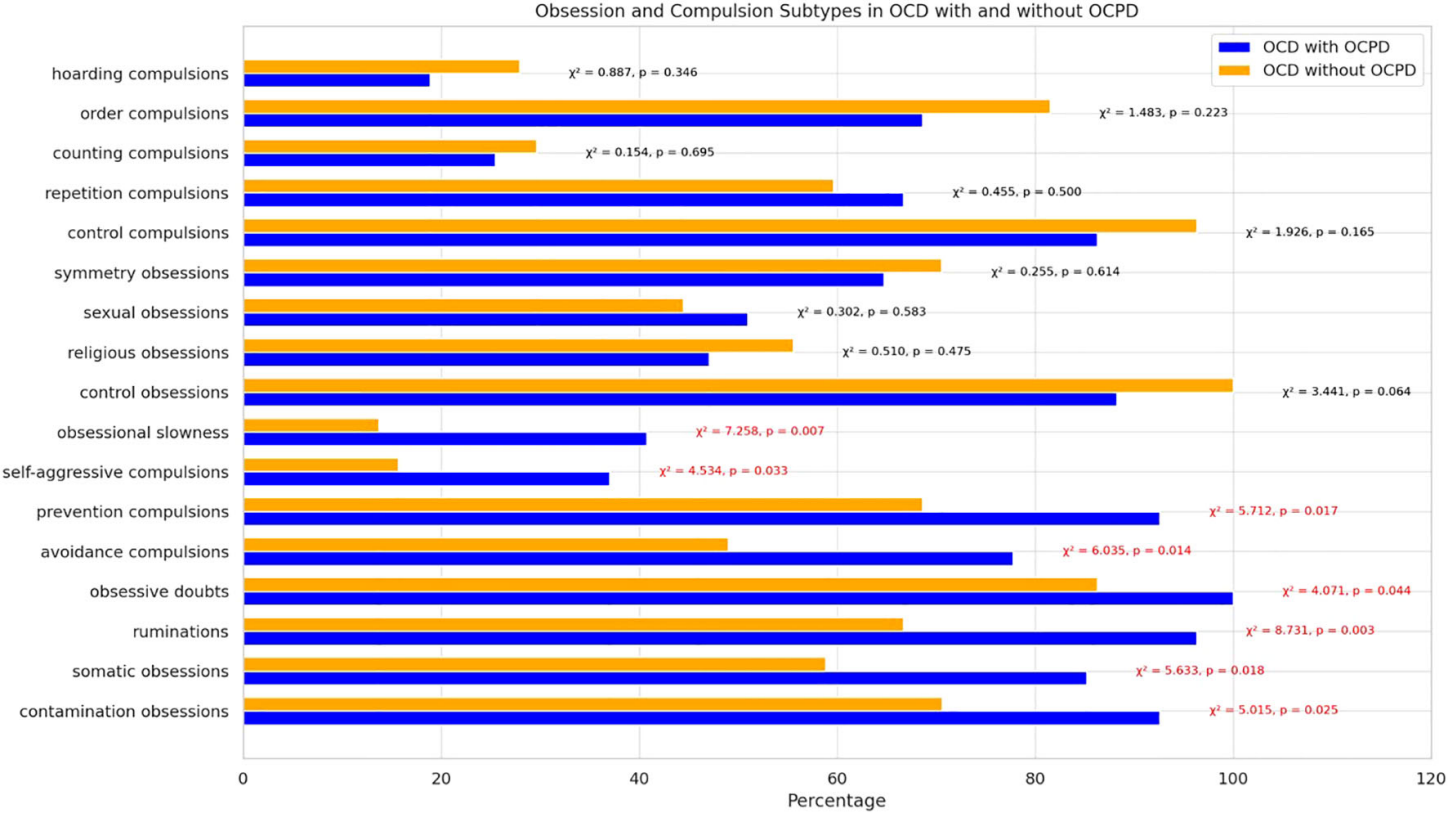
Distribution and comparison of the most common obsessions and compulsions in OCD and OCD with OCPD subgroups based on Y-BOCS results.

The chi-square test evaluating the association between distinct obsessive-compulsive symptom dimensions and the presence of OCPD revealed significant differences between patients with OCD alone and those with both OCD and OCPD. In the OCD with OCPD group, significant differences were observed for the following symptoms: contamination obsessions (92.59% vs. 70.59%, chi-square = 5.015, p = 0.025), somatic obsessions (85.19% vs. 58.82%, chi-square = 5.633, p = 0.018), ruminations (96.30% vs. 66.67%, chi-square = 8.731, p = 0.003), obsessive doubts (100% vs. 86.27%, chi-square = 4.071, p = 0.044), avoidance compulsions (77.78% vs. 49.02%, chi-square = 6.035, p = 0.014), prevention compulsions (92.59% vs. 68.63%, chi-square = 5.712, p = 0.017), self-aggressive compulsions (37.04% vs. 15.69%, chi-square = 4.534, p = 0.033), and obsessional slowness (40.74% vs. 13.73%, chi-square = 7.258, p = 0.007). Furthermore, one obsession subtype, specifically sexual obsessions, was observed more frequently in the OCD with OCPD group compared to those with OCD alone, though this difference did not reach statistical significance (50.98% vs. 44.44%, chi-square = 0.302, p = 0.583). In the OCD alone group, while no statistically significant differences were found, several obsession and compulsion subtypes were more frequently observed compared to the group with both OCD and OCPD: hoarding obsessions (68.63% vs. 18.52%, chi-square = 19.194, p < 0.001), hoarding compulsions (68.63% vs. 29.63%, chi-square = 11.051, p = 0.001), control obsessions (100% vs. 88.24%, chi-square = 3.441, p = 0.064), religious obsessions (55.56% vs. 47.06%, chi-square = 0.510, p = 0.475), control compulsions (96.30% vs. 86.27%, chi-square = 1.926, p = 0.165), repetition compulsions (74.07% vs. 66.67%, chi-square = 0.455, p = 0.500), counting compulsions (29.63% vs. 25.49%, chi-square = 0.154, p = 0.695), and order compulsions (81.48% vs. 68.63%, chi-square = 1.483, p = 0.223). Key differences are shown in **Figure 2**.

## 5 Discussion

The phenomenon of anankastic personality is still a poorly understood and underestimated problem (40–42, 45, 98). However, the results of our study showed that the co-occurrence of OCD with OCPD is relatively common, which was found in 34.61% of patients in the entire study group. This result confirms the findings obtained by other researchers (42, 53, 54, 98). Furthermore, our study demonstrated that the presence of OCPD significantly affects several critical aspects of OCD. In particular, it exacerbates the severity of OC symptoms and worsens insight into obsessive-compulsive behavior. These findings are also consistent with those reported by other researchers (42, 53, 54, 57). Greater severity of OCD symptoms, accompanied by significantly poorer insight and resulting in ego-syntonic obsessions, has been shown to negatively impact the course of treatment (24, 99, 100). This highlights that ego-syntonicity is associated with reduced motivation and poor treatment adherence (101, 102). Consequently, it is not surprising that the group with both OCD and OCPD had a significantly longer treatment duration (13.185 years) compared to those without OCPD (7.235 years). Our findings indirectly correlate with a longitudinal study by Eisen et al. (102), which found that OCPD comorbidity was associated with more than twice the risk of OCD recurrence after remission over a five-year follow-up period (103).

Comparing the two study subgroups, no significant differences were observed in terms of age, education, and the ability to maintain close relationships. However, among patients with both OCD and OCPD, 67.74% were unemployed compared to 44.90% without OCPD, and they were significantly older than respondents with OCD alone (47.767 and 40.730 years old, respectively). These results, supported by Diedrich and Voderholzer (41), confirm that the presence of OCPD negatively impacts the course of OCD and reduces life satisfaction due to significantly lower sexual functionality (41). These findings are also confirmed by a study in which poorer quality of life and the presence of depression were considered predictors of OCD course severity (99). Thus, as described by Jaisoorya and Janardhan (21), the problem with functional recovery of OCD patients can be explained by the frequent co-occurrence of OCPD (21). Furthermore, the significantly reduced sexual functionality observed in patients with a dual diagnosis may correspond with other findings from our research, such as the increased levels of aggression and impulsivity, which were also found to be significantly higher in subjects with both OCD and OCPD. These results may relate to findings from a study on the severity of impulsivity and aggression among psychiatric patients, where 24-52% of the 118 subjects exhibited features of OCPD (40). In individuals with OCD, elevated anger and impulsivity have been noted to worsen OC symptoms, contributing to increased depression, reduced insight, and the development of dysfunctional beliefs and maladaptive emotion regulation strategies (104–107). These observations suggest that the presence of OCPD, with heightened aggressive impulsivity, may complicate the therapeutic process and consequently prolong treatment duration, as demonstrated in our study, potentially serving as a predictor of poorer outcomes in OCD.

Equally interesting was the demonstration that OCPD contributes to significantly increased levels of depression and mania. It has been confirmed that affective disorders worsen the course of OCD and negatively affect the patient’s quality of life (18, 26, 99, 108, 109). As described in the introduction, a study by Hantouche et al. (109) suggests a distinct form of OCD, referred to as cyclothymic OCD (110). More specifically, it is characterized by the following features: a higher number of manic/hypomanic episodes and major depressive episodes, a higher frequency of aggressive, impulsive, religious, and sexual obsessions, control compulsions, hoarding, repetition; and finally, an elevated rate of mood changes with aggressive behavior (77). Interestingly, despite these findings, Hantouche et al. did not explore the potential impact of OCPD in their conclusions. However, our patients with OCD and OCPD shared very similar phenomena to those reported above: greater symptom severity, higher levels of mania and depression, and increased levels of impulsivity and aggression. In turn, the demonstrated and elevated frequency of obsessive doubts, avoidance and prevention OC may be related to the need for control and repetition described in the by Hantouche’s study. Therefore, it can be hypothesized that,cyclothymic OCD’ is in fact an obsessive-compulsive disorder with a comorbid anankastic personality. In turn, the previously cited Rossi et al. (87) and Altindag (88) both highlighted the prevalence of OCPD in patients with BD (19 - 32%) (88, 89). As our study demonstrated significantly elevated levels of depression and mania in patients with both OCD and OCPD, this suggests a broader connection between these disorders and bipolarity, potentially explaining the higher prevalence of BD in OCD, estimated at 11% to 35%, which is similar to the comorbidity rates of OCD with OCPD, ranging from 15% to 36% (29, 30, 42, 53–56, 78, 111–113).

In our study, we also aimed to assess the strength of correlations between statistically significant variables showed in the group with OCPD and their potential impact on bipolarity.

Significant positive correlations were observed between depressive symptoms (HDRS) and overall aggression (BP-total, p = 0.353), anger (BP-A, p = 0.400), hostility (BP-H, p = 0.322), as well as OCD severity (Y-BOCS total, p = 0.421; Y-BOCS obsessions, p = 0.378; Y-BOCS compulsions, p = 0.423). These findings indicate that passive forms of aggression and higher OCD severity are closely linked to depression in this group. No significant correlations were found between impulsivity or aggression and mania severity. Based on these results, the predictors of bipolarity in the OCD with OCPD may include heightened depressive symptoms, aggression (particularly anger and hostility), and increased OC severity. This highlights the need for further investigation into the relationship between OCPD and bipolarity in OCD. Notably, in the meta-analysis by Amerio et al. (79), which explored the prevalence and predictors of comorbid BD and OCD, OCPD was not mentioned (79). Similarly, in the more recent meta-analysis by de Filippis et al. (113), which framed OCD as a possible epiphenomenon of comorbid bipolar disorder, OCPD was also not considered (114).

In our study, individuals with both OCD and OCPD exhibited significantly higher rates of contamination, somatization, doubt, and rumination obsessions, alongside avoidance, prevention, and self-aggression compulsions. This may contribute to a more unfavorable course of OCD, particularly by exacerbating obsessional slowness. The group with OCPD showed a significantly stronger association with this phenomenon, rarely described in the medical literature, characterized by extreme helplessness during obsessions and avoidance of activities due to the fear of compulsions lasting many hours or even days (6, 60, 74, 115). In contrast, patients with OCD alone showed higher, though not statistically significant, rates of hoarding, control, religious, and symmetry-related obsessions, as well as repetition, counting, and ordering compulsions. Our study included a relatively small sample size, which may account for some of the variations observed compared to the findings of Gordon et al. (58) and Thamby and Khanna (55), where hoarding, symmetry, and ordering symptoms were more prominent in cases of comorbid OCPD. These discrepancies may also reflect differences in methodology or patient populations (55, 58).

In analyzing the identified OC symptoms, it is worth revisiting the influence of OCPD on bipolarity within OCD. Unlike other studies, De Prisco et al. (115), through a meta-analysis, demonstrated that only two OC symptoms - sexual and contamination obsessions - were significant in OCPD-BD comorbidity (116). In our sample of individuals with both OCD and OCPD, we also observed an increased presence of contamination and somatic obsessions, though only contamination obsessions reached statistical significance. These findings suggest that contamination obsessions may serve as indicators of OCPD’s influence on bipolarity within OCD, highlighting the need for further investigation into this relationship.

Reflecting on all the deficits observed due to the presence of OCPD in the course of OCD, this co-occurrence proves to have a significantly negative impact. OCPD exacerbates OCD symptoms by increasing the severity of OC, contributing to obsessive slowness, and leading to reduced insight. Moreover, patients with coexisting OCPD tend to be older, require longer treatment durations, and exhibit increased levels of impulsivity, further complicating the course of OCD. In addition, our analysis revealed that OCPD significantly impacts levels of both depression and mania, with heightened depressive symptoms correlating with increased aggression (anger and hostility), and more severe OCD symptoms, such as obsessive-compulsive behaviors, serving as potential predictors of bipolarity in this group.

Taken together, our findings point to the potential relevance of comorbid OCPD in shaping the clinical course and symptom profile of OCD. Further research based on larger and more diverse clinical samples is needed to confirm these associations, particularly in relation to bipolar features and treatment responsiveness. These preliminary observations may offer a useful starting point for developing more individualized clinical approaches.

## 6 Conclusions

1. The presence of OCPD is relatively common in individuals with OCD.
2. OCPD in the context of OCD can be considered a predictor of increased OCD severity and reduced insight, being significantly associated with older age.
3. The comorbidity of OCPD with OCD significantly increases levels of depression and mania in individuals with OCD.
4. The influence of OCPD on OCD results in a significant increase in levels of aggression and impulsivity.
5. The presence of OCPD is associated with higher rates of contamination and somatic obsessions, ruminations, avoidance and prevention compulsions, and obsessional slowness.
6. Predictors of bipolarity in OCD with comorbid OCPD include heightened depressive symptoms, aggressive impulses, and obsessive-compulsive symptoms.
7. The presence of OCPD may be associated with factors such as unemployment and reduced quality of sexual life in individuals with OCD.
8. Diagnosing OCPD should be a routine part of assessing patients with OCD.
9. The interactions between OCPD, OCD, and bipolarity should be explored in future studies.

## 7 Limitations of the study

- The results for assessing the prevalence of individual obsessions and compulsions in both subgroups were based on small patient groups, which could potentially bias the final assessment and limit the generalizability of the findings.
- Only selected phenomena were included in the comparison of both subgroups, suggesting that other variables not covered in the study might differentiate or further align patients with OCD and OCPD.
- The study did not take into account the diversity of treatment approaches, focusing primarily on a sample in which 93.6% of participants received pharmacological treatment (alone or in combination with psychotherapy). As a result, it was not possible to assess the potential differential effects of treatment modalities on the clinical course of OCD in patients with comorbid OCPD.
- Retrospective data on the age of OCD onset were not collected, which limits the ability to reconstruct the chronological development of symptoms. In the case of OCPD, the gradual and often diffuse nature of personality trait formation makes determining a specific onset point inherently difficult.
- The assessment of OCPD may be influenced by the presence of active OCD symptoms. Although diagnoses were based on clinical judgment and DSM-5 criteria, evaluating OCPD during symptom remission might increase diagnostic accuracy.

## Data Availability

The raw data supporting the conclusions of this article will be made available by the authors, without undue reservation.
